# PCB Exposure in Adult Male Mice Reduces Proliferating Cells in the Prostate but Minimally Alters Voiding

**DOI:** 10.3390/toxics14030265

**Published:** 2026-03-18

**Authors:** Kathy Wang, Audrey Spiegelhoff, Tamryn Jordan, Thomas Lavery, Conner L. Kennedy, Monica M. Ridlon, Kimberly P. Keil Stietz

**Affiliations:** Department of Comparative Biosciences, School of Veterinary Medicine, University of Wisconsin-Madison, Madison, WI 53706, USA

**Keywords:** void spot assay, cystometry, LUTS, environmental exposure

## Abstract

Lower urinary tract dysfunction (LUTD) is a multifactorial disease process that encompasses diverse symptoms ranging from issues with storage and sensation to impaired emptying of the bladder. Furthermore, symptoms tend to worsen with age and other comorbidities and in men can also be influenced by changes to the prostate, making diagnosis and treatment difficult to manage. Environmental factors are thought to contribute to disease risk. In mice, previous work has found that developmental exposure to polychlorinated biphenyls (PCBs) is capable of altering voiding function in offspring. However, the effects of PCB exposure in adulthood instead of development are not well known. Whether changes in voiding are a consequence of early or later life exposures remains an important area of study, as environmental chemicals and exposures can occur across the lifespan and can be mitigated. Here, we test whether PCB exposure in adulthood alters voiding or prostate morphology in male mice. C57Bl/6J adult male mice were exposed to the human-relevant MARBLES PCB mixture (0, 0.1, 1, and 6 mg/kg/d) orally daily for two months. Lower urinary tract function was then assessed through urodynamic testing including void spot assay, uroflowmetry, and anesthetized cystometry. Prostate lobes were collected for histology. The only change to voiding function was a reduction in void duration in the 6 versus 1 mg/kg/d PCB group but not to the vehicle control. Prostate, seminal vesicle, and testes wet weights were unchanged. However, PCB exposure reduced the number of Ki67-positive proliferating cells in the anterior and ventral prostate lobes only at the 1 mg/kg/d dose, with no change to caspase 3-positive cells or smooth muscle thickness. Together, these data indicate that 2-month exposure to PCBs in adult mice has little impact on voiding but is a sufficient exposure to produce changes in cell proliferation in the prostate. The mechanistic impacts of these changes remains to be investigated but could help better understand individual risk for LUTD.

## 1. Introduction

Exposure to exogenous chemicals occurs throughout life, yet the impacts of these exposures on the lower urinary tract, consequences of timing, and mechanisms of action remain poorly understood. Lower urinary tract dysfunction (LUTD) is a prevalent and pervasive health concern whose symptoms greatly impact quality of life [1]. In a meta-analysis including 222 studies through the year 2021, the study estimates global prevalence of any (mild to severe) lower urinary tract symptoms (LUTS) to be 63.2% [2]. Moderate to severe LUTS were more prevalent in men than women with an estimated prevalence of 35.2% and 20.6%, respectively [2]. Therefore, it is of interest to better understand the impacts of modifiable risk factors, such as exogenous chemicals, to inform strategies of prevention and treatment, which may help to mitigate or reduce LUTD and improve human health.

Polychlorinated biphenyls (PCBs) are persistent organic pollutants that are broadly detected in human and animal tissues [3,4,5,6,7,8,9,10,11,12,13,14]. Although most intentional production has been banned, legacy sources of PCBs remain in the environment due to their stability and long half-lives [15]. Concerningly, contemporary sources of PCBs are generated unintentionally as byproducts of several processes, including pigment production [14,16,17]. As a result, human exposure to PCBs persists [15,18]. Further, several contemporary PCBs and their metabolites, many of which are less well studied, are increasingly detected in human tissues, including in serum of pregnant women and brain tissue [4,15,18,19,20]. PCBs are associated with multiple adverse health outcomes including endocrine disruption [21,22,23], immune dysfunction [24,25,26,27,28,29,30,31,32], and neurotoxicity [4,33,34,35,36,37,38]. However, their effects on the lower urinary tract have only recently begun to be explored [39,40,41]. 

Previous studies in mice demonstrate that in utero and lactational exposure to an environmentally relevant contemporary mixture of PCBs (termed the MARBLES PCB mixture [19,42]) leads to changes in lower urinary tract function of the resulting young adult male offspring, including increased quantity of small drops like void events and increased peak intravesical pressure during voiding [40]. Developmental PCB exposure increases immune cells [43] and nerve fiber density [44] in the bladder of juvenile mouse offspring. Adulthood PCB exposure in female mice has been shown to have subtle effects on voiding function and bladder contractility [45], yet the effects of adult PCB exposure on male voiding function has not been examined and is the goal of this study.

A subset of LUTS in men can be associated with prostate enlargement or morphological changes, leading clinically to a diagnosis of benign prostatic hyperplasia (BPH). Hallmarks of BPH-associated LUTS typically include difficulty voiding, straining, and weak stream [46]. Therefore, when assessing voiding function in male mice, it is important to consider effects of the prostate. The prostate is a hormonally sensitive organ [47], and environmental endocrine-disrupting chemicals, which can mimic endogenous steroid hormones, have been shown to alter prostate mass and voiding function [48,49]. Several PCBs and their metabolites can also act as endocrine disruptors [15,21,50,51]. Interestingly, developmental MARBLES PCB exposure leads to male mice at 6 weeks of age exhibiting a reduction in ventral prostate mass in the middle versus low and high PCB dose groups [41]; interestingly, at 12 weeks of age this effect was still observed but with the middle and highest PCB dose group having reduced prostate mass compared with the lowest PCB group [52]. Since changes in prostate mass can alter voiding and since chemicals can alter prostate mass, another goal of this study was to determine whether adult MARBLES PCB exposure influences the prostate, with or without accompanying changes in voiding.

Given that humans continue to be exposed to PCBs throughout life, combined with the fact that LUTS prevalence increases with age [2], we sought to test the hypothesis that adult PCB exposure alters voiding function and prostate morphology in male mice. We found few effects of a two-month exposure to a mixture of PCBs on voiding function in adult males; however, PCB exposure decreased prostate proliferation at the medium but not low or high dose, indicating that this exposure window in adult mice is sufficient to induce measurable changes in prostate cells while producing minimal effects on voiding at this stage. These findings raise the possibility that these changes may progress with continued exposure or in combination with additional age-related stressors and warrant further study.

## 2. Materials and Methods

### 2.1. Animals

All procedures involving animals were conducted in accordance with the NIH Guide for the Care and Use of Laboratory Animals and were approved by the University of Wisconsin-Madison Animal Care and Use Committee. C57Bl/6J wild-type mice were purchased from Jackson Labs (Bar Harbor, ME, USA) and subsequently bred in-house. Adult male mice were randomly selected to treatment groups. Each treatment group consisted of animals generated from 6–7 litters. Mice were either singly housed or housed with another mouse of the same treatment group, which was balanced across groups. Body mass of the mice at the start of the study did not differ between assigned groups (mean ± SEM are as follows: vehicle 25.8 g ± 0.84, 0.1 mg/kg 25.2 g ± 0.77, 1 mg/kg 25.7 g ± 0.47, 6 mg/kg 25.3 g ± 0.65, F (3,28) = 0.1997 *p* = 0.8957). All mice were housed in clear plastic cages containing corn cob bedding and maintained on a twelve-hour light and dark cycle at 22 ± 2 °C with relative humidity levels ranging between 30% to 70%. Feed (Diet 2020x, Teklad, Indianapolis, IN, USA) and water were available ad libitum.

### 2.2. PCB Exposure

PCBs were obtained from Accustandard (Accustandard, New Haven, CT, USA). Mice were dosed as described [44,45]. Briefly, 10-week-old adult male mice (average age 70.6 ± 1.3 days old), n = 8 per group, were dosed orally daily at concentrations of 0, 0.1, 1, or 6 mg/kg MARBLES PCB mixture. PCBs were dissolved in peanut oil (Spectrum Organic Products, LLC, Mellville, NY, USA) and mixed into organic peanut butter (Trader Joe’s, Monrovia, CA, USA). A 20 mg/mL PCB stock in peanut oil was diluted into peanut butter at 0 (oil only), 0.025, 0.25, and 1.5 mgPCB/g peanut butter, respectively, and these stocks were used to administer doses based on mouse mass. Mice were singly housed during dosing and were observed to ensure consumption. The MARBLES PCB mixture and these doses have been defined and used previously to produce changes in voiding function in adult female mice [45] and developmentally exposed male and female offspring [40] and result in relevant dose-dependent tissue levels in mice [44,53,54]. Mice were dosed for 2 months (average days dosed 60.8 ± 1.6 days) to mimic the dosing period of adult female dams in a previous study [45] and to accommodate for the ability to run voiding behavior assays on subsequent days. Adult males then underwent void spot assay (VSA), uroflowmetry, and anesthetized cystometry as described below and were euthanized, with CO_2._ Following euthanasia, tissues were dissected, weighed and collected. The average age of all animals at time of tissue collection was 131.5 ± 1.4 days old.

### 2.3. Immunohistochemistry

Following euthanasia, testes were removed and weighed. The lower urinary tract was removed and seminal vesicle and prostate lobes were micro-dissected (anterior, dorsolateral, and ventral) and weighed. Tissues were fixed in 4% paraformaldehyde (Fisher, Madison, WI, USA) overnight, dehydrated into 100% methanol for long-term storage, and processed into paraffin blocks as described previously [44,55]. Immunohistochemistry was performed as described [55] using the antibodies and dilutions listed in Table 1. An Eclipse Ci compound microscope fitted with a DS Ri2 camera (Nikon Instruments Inc., New York, NY, USA) with NIS elements imaging software (D 5.02.01) (Nikon Instruments Inc., New York, NY, USA) was used for imaging. For fluorescent images, areas to image were identified based on DAPI (nuclei) or the epithelial marker, E-Cadherin (CDH1), to eliminate bias. Exposure settings were kept consistent across groups on the slide. Quantification of endpoints was performed by an experimenter blinded to treatment conditions. Ki67- and cleaved caspase 3 (Casp3)-positive cells were quantified using the Image J counter feature. Ki67- or Casp3-positive cells were expressed as a percentage of total cells (DAPI+) counted within the epithelium and the stroma of the image. Prostate smooth muscle (ACTA2+) thickness was quantified using the Image J (v.1.46r) measure feature by taking multiple thickness measures (at least 5) per image of the smooth muscle ACTA2+ layer around individual ducts. Multiple images were taken per tissue for analysis from an *n* = 4–5 mice per treatment group. Final measures were averaged per tissue such that the animal was the *n* value for statistics. All *n* values are also reported in the respective figure legends.

### 2.4. Void Spot Assay (VSA)

Void spot assays were conducted according to best practices as described [56,57]. Briefly, mice were acclimated to the quiet room for 1 h. Mice (n = 8 mice per treatment group) were placed singly in an empty cage fitted with a 3 MM chromatography paper (057163E, Thermo Fisher Scientific, Waltham, MA, USA) cut to the size of the cage bottom. The mice had access to food, but not water, during the 4 h testing period. Testing was conducted during the light phase beginning at ~10 AM. After testing, chromatography papers were allowed to dry and were imaged using a UVP ChemStudio Plus UV imager (Analytik Jena, Beverly, MA, USA). VSA analysis was conducted in Image J (v.1.46r) using the open-access Void Whizzard analysis plugin for Image J [56] by an individual blinded to treatment conditions.

### 2.5. Uroflowmetry

Uroflowmetry was conducted as previously described [40,58]. At least one day following VSA testing, mice (n = 8 mice per treatment group) were acclimated to the room for 1 h and then placed into uroflowmetry chambers for 4 h with access to water, but not food. Uroflow data was collected using Raspberry Pi cameras and processors (RS Components Limited, Corby, UK) as described [58]. Data was analyzed by an individual blinded to treatments. Urine events in which urine hit the bars of the metabolic chamber’s floor were excluded from analysis, which led to a final n value of 6, 8, 8, 7 for each treatment group, respectively. A scale from 1 to 3 was used to determine a numerical value for stream rating, in which 1 was a void of individual drops and 3 was a void of a strong stream of urine.

### 2.6. Anesthetized Cystometry

Anesthetized cystometry was conducted as described [40,59]. Briefly, mice (n = 8 mice per treatment group) were anesthetized using a subcutaneous injection of urethane (AC325540500, Thermo Fisher Scientific, Waltham, MA, USA) at a dosage of 1.43 g/kg, which was given from a fresh urethane stock of 0.086 g/mL in saline. A PE-50 tubing catheter (NC9140178, Thermo Fisher Scientific, Waltham, MA, USA) was placed in the bladder dome. Following surgery, mice were allowed to recover for approximately 60 min on a heated pad and then connected via the catheter to an in-line pressure transducer and infusion pump. Saline was infused into the bladder at a rate of 0.8 mL/hr a standard rate in mouse cystometry studies [40,59]. Bladder pressure was recorded using an MLT844 physiological pressure transducer (ADInstruments, Colorado Springs, CO, USA) connected to an FE221 Bridge Amp (ADInstruments, Colorado Springs, CO, USA) with a PowerLab 2/26 (PL2602) data acquisition system. Cystometrograms were recorded and analyzed using Labchart software (v8.1.30) (ADInstruments, Colorado Springs, CO, USA). Cystometrograms were recorded for approximately 1 h, or until a consistent pattern of voiding was achieved. A total of 5 consecutive voids for each mouse were averaged and analyzed for data collection and selected by an individual blinded to treatment conditions. Measured parameters include intervoid interval, void duration, normalized threshold pressure, normalized max voiding pressure, non-voiding contractions, and compliance. If an animal failed to elicit voids or the catheter leaked or did not remain in the bladder, that animal was excluded from analysis resulting in a final n = 7, 7, 6, 8 per treatment group, respectively.

### 2.7. Statistics

Statistical analysis was conducted using GraphPad PRISM 10. Normality was assessed using Shapiro–Wilk and Kolmogorov–Smirnov tests. Variance was assessed using Bartlett’s test or Brown-Forsythe’s test. If normality and variance assumptions were met, data were analyzed using a one-way ANOVA followed by Tukey’s or Dunnett’s multiple comparisons tests. If data failed to meet normality assumptions, data were analyzed using a Kruskal–Wallis test followed by Dunn’s multiple comparisons test. A two-way repeated measures ANOVA test was conducted on void spot assay spot size distribution data. Fisher’s exact test was used to assess percentage of mice with specified urine spot numbers in Table 2. One outlier was removed from the dorsal prostate mass data as confirmed by the Prism ROUT method (Q = 1%) to test for outliers. *p*-values ≤ 0.05 were considered significant. All *n* values are also indicated in the figure legends along with the specified statistical tests used.

## 3. Results

### 3.1. PCB Doses Influence Void Duration

**Figure 1:**
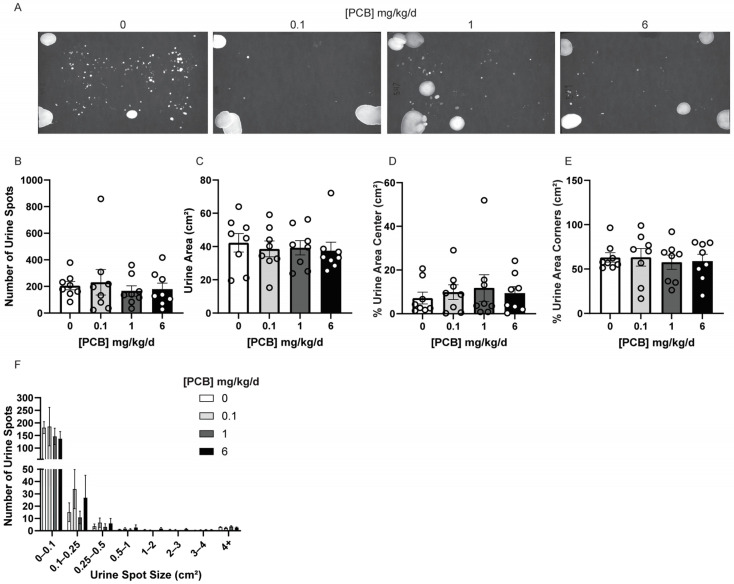
Adult PCB exposure does not alter the total number of urine spots produced during VSA. Adult male mice were exposed to a daily dosage of the MARBLES PCB mixture for 2 months and underwent VSA testing. (**A**) Representative images of VSA. VSA testing parameters include (**B**) total number of urine spots, (**C**) total urine area (cm^2^), (**D**) percent urine area in center of the paper (cm^2^), (**E**) percent urine area in the corners of the paper (cm^2^), and (**F**) urine spot size distribution (cm^2^). Results are mean +/− SEM n = 8 mice/group. No significant differences as determined by a one-way ANOVA test (**B**,**D**,**E**), Kruskal–Wallis test (**C**), and two-way repeated measures ANOVA test followed by Tukey’s multiple comparisons test (**F**).

In order to assess conscious voiding patterns in adult male mice, VSA was performed. There was no effect of PCBs on the number of urine spots, urine area, or the percentage of urine in the center or corners of paper (Figure 1A–E). Urine spots were also assessed by size, and no significant PCB effects were found on spot size distributions (Figure 1F). Since the presence of a ‘frequent spotting’ phenotype can be present [57], especially in male mice, we also assessed the percentage of mice in each treatment group, which produced more than 50, 100, 200, 300, or 400 urine spots during the 4 h testing period (Table 2). The percentage of mice in each spot number category did not significantly differ from the control. A trend was observed in that the 0.1 and 6 mg/kg/d PCB treatment groups were the only groups to have mice which had over 400 total spots at 12.5% of mice compared with none observed in the vehicle or 1 mg/kg/d PCB treatment groups (Table 2).

**Figure 2:**
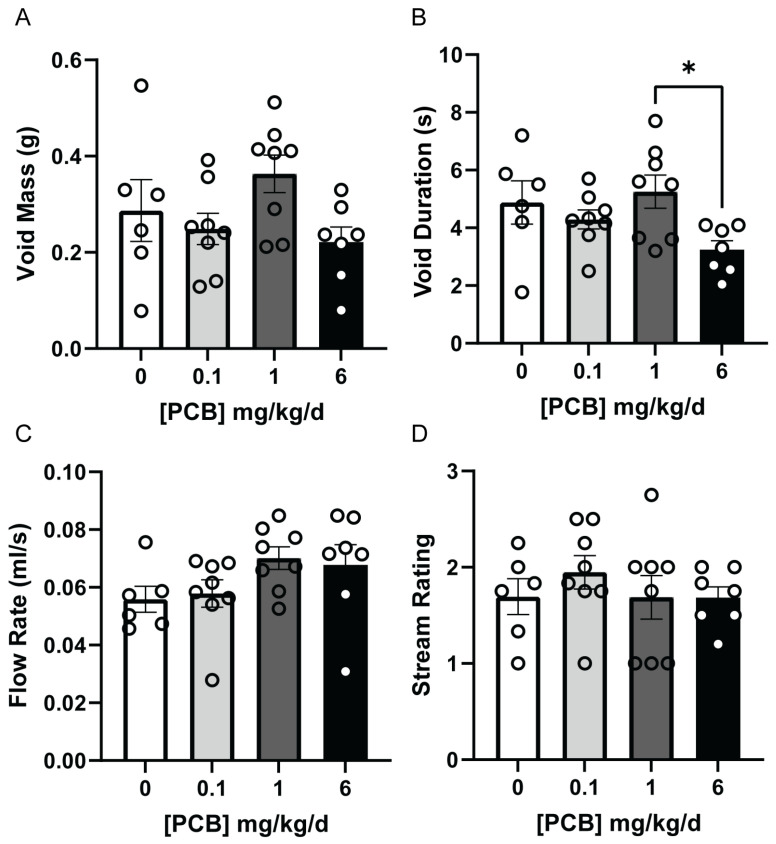
Adult PCB exposure alters void duration between the 1 and 6 mg/kg/d dosage groups. Adult male mice were exposed to a daily dosage of the MARBLES PCB mixture for 2 months and underwent uroflowmetry testing to examine parameters of (**A**) void mass (g), (**B**) void duration (s), (**C**) flow rate (mL/s), and (**D**) stream rating. Results are mean ± SEM n = 6–8 mice/group. * and bar indicate significant differences *p* ≤ 0.05, as determined by a one-way ANOVA test followed by Tukey’s multiple comparisons test.

Since VSA can only capture void events over an entire testing period, we also assessed voiding using uroflowmetry. Uroflowmetry is valuable as it recapitulates testing performed on humans to determine changes in flow rate. The average mass of voids did not differ between treatment groups (Figure 2A), but the void duration was significantly decreased in the 6 mg/kg/d PCB group versus the 1 mg/kg/d PCB group (Figure 2B; F(3,25) = 3.052, *p* = 0.0470, 1 vs. 6 *p* = 0.0377). However, there were no significant changes to flow rate or stream rating (Figure 2C,D).

**Figure 3:**
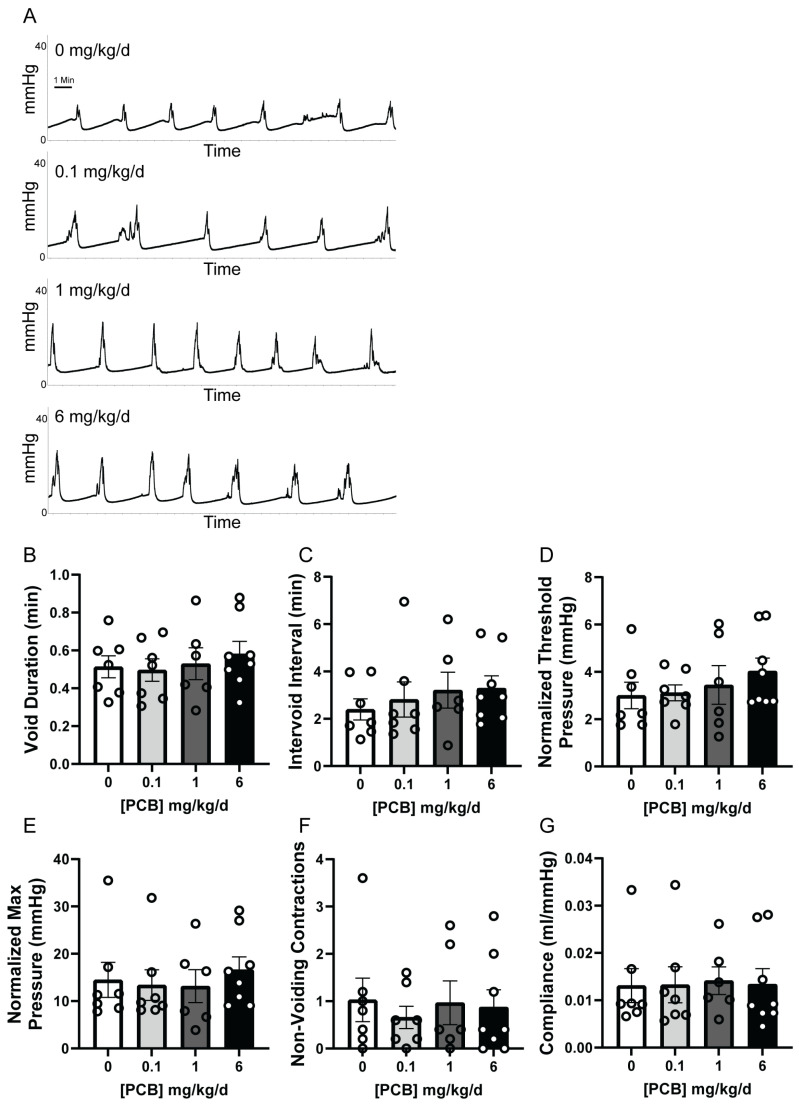
Adult PCB exposure does not alter cystometry voiding metrics. Adult male mice were exposed to a daily dosage of the MARBLES PCB mixture for 2 months and underwent anesthetized cystometry. (**A**) representative cystometrograms. Analysis of (**B**) void duration (min), (**C**) intervoid interval (min), (**D**) normalized threshold pressure, (**E**) normalized maximum pressure, (**F**) non-voiding contractions, and (**G**) compliance (ml/mmHg). Results are mean ± SEM n = 6–8 mice/group. No significant differences *p* ≤ 0.05, as determined by a one-way ANOVA test (**B**,**D**) or Kruskal–Wallis test (**C**,**E**–**G**).

In order to assess bladder filling mechanics and remove behavioral stimuli, anesthetized cystometry was conducted. There were no significant differences in void duration, void interval, threshold pressure, maximum pressure, non-voiding contractions, or compliance (Figure 3A–G). Together, these data indicate that there are no functional changes to adult male voiding patterns following adult PCB exposure compared with the vehicle control. However, a few differences were seen between PCB doses for void duration.

### 3.2. PCBs Do Not Alter Prostate Mass but Reduce Number of Ki-67-Positive Cells

**Figure 4:**
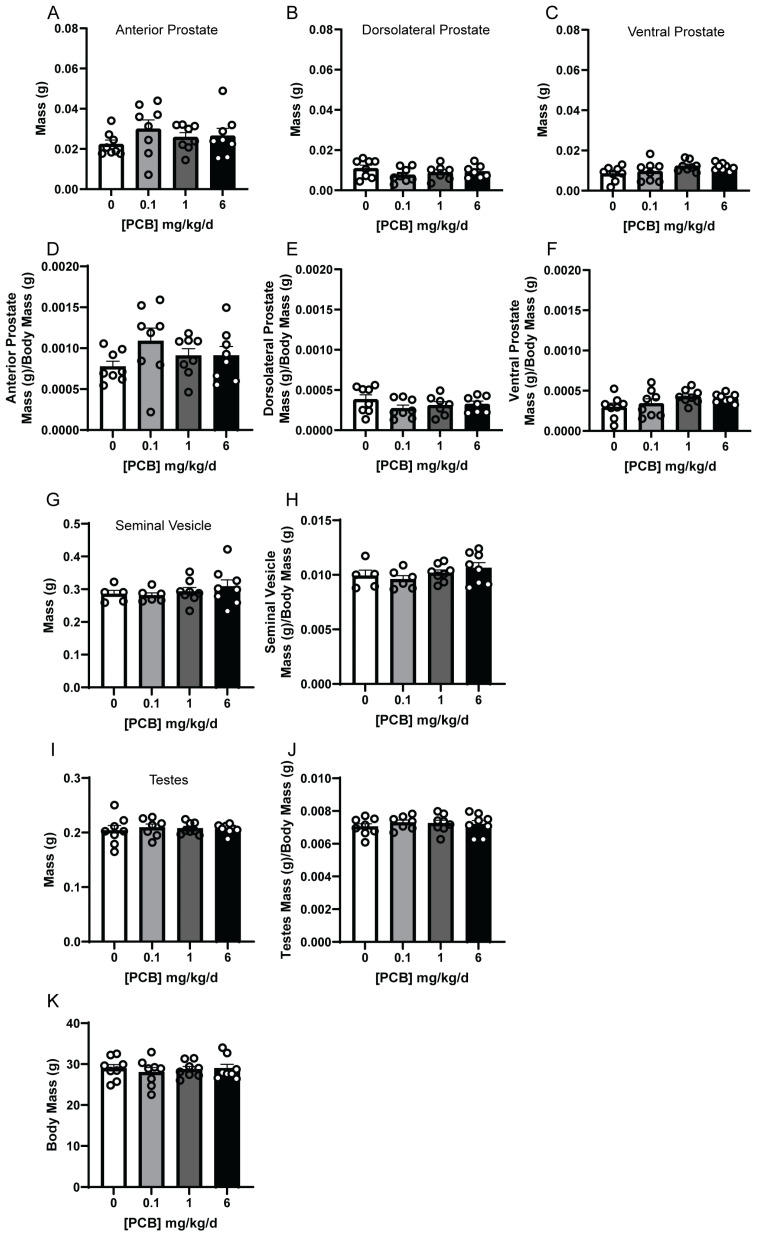
Adult PCB exposure does not change prostate mass. Adult male mice were exposed to a daily dosage of the MARBLES PCB mixture for 2 months and tissues collected. Quantification of (**A**) anterior prostate mass, (**B**) dorsolateral prostate mass, (**C**) ventral prostate mass, (**D**) anterior prostate mass normalized to body mass, (**E**) dorsolateral prostate mass normalized to body mass, (**F**) ventral prostate mass normalized to body mass, (**G**) seminal vesicle mass, (**H**) seminal vesicle mass normalized to body mass, (**I**) testes mass, (**J**) testes mass normalized to body mass, and (**K**) body mass. Results are mean ± SEM, n = 7–8 per treatment group, n = 5–8 for seminal vesicle mass. No significant differences *p* ≤ 0.05, as determined by a one-way ANOVA test. One outlier was identified in Prism and removed in the dorsal prostate 6 mg data (**B**,**E**).

We have previously found that developmental exposure to PCBs can influence the mass of prostate lobes in young adult offspring at both 6 and 12 weeks of age [41,52]. Here, we wanted to further examine whether adult exposure was sufficient to alter prostate mass. There were no significant effects of PCBs on prostate lobe wet weights alone or when normalized to body mass (Figure 4A–F). There were also no changes in seminal vesicle, testes, or body mass (Figure 4G–K). These results indicate that the mass of these hormone-sensitive tissues is not altered at this timepoint.

**Figure 5:**
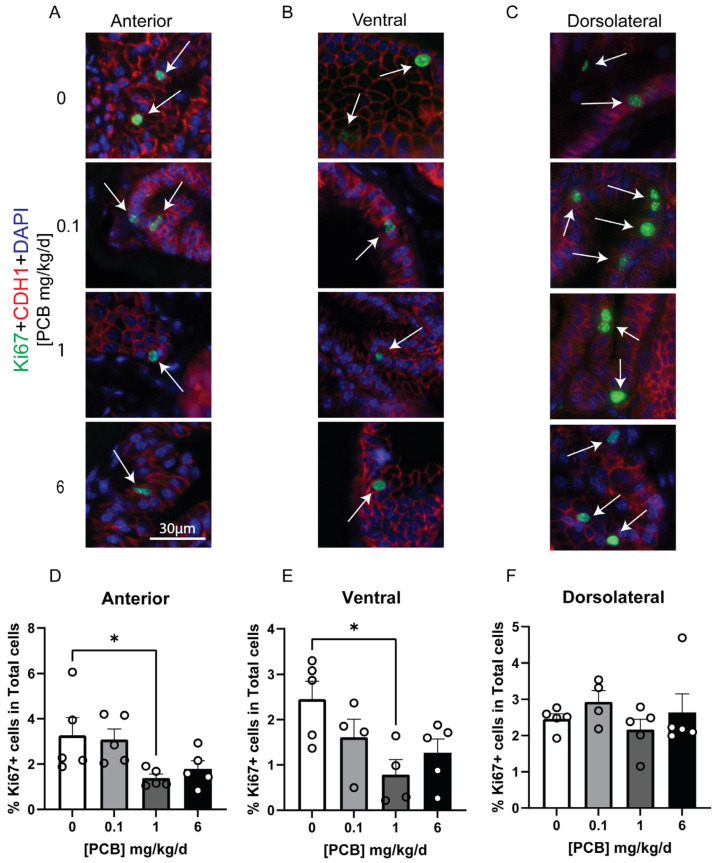
Adult PCB exposure decreases KI67-positive cells in the anterior and ventral prostate. Adult male mice were exposed to a daily dosage of the MARBLES PCB mixture for 2 months and tissues collected for histology. Representative images of (**A**) anterior prostate, (**B**) ventral prostate, and (**C**) dorsolateral prostate stained with antibodies targeting Ki67 (green) and E-cadherin (Red, all epithelium) and counterstained with DAPI to label all nuclei (blue). Quantification of the percentage of total cells which are Ki67+ in the (**D**) anterior prostate, (**E**) ventral prostate, and (**F**) dorsolateral prostate. Arrows indicate positive cells. Results are mean ± SEM, n = 4–5 per treatment group. * and bar indicate significant differences *p* ≤ 0.05, as determined by a one-way ANOVA test followed by Dunnett’s multiple comparisons test (**D**,**E**) or a Kruskal–Wallis test (**F**).

**Figure 6:**
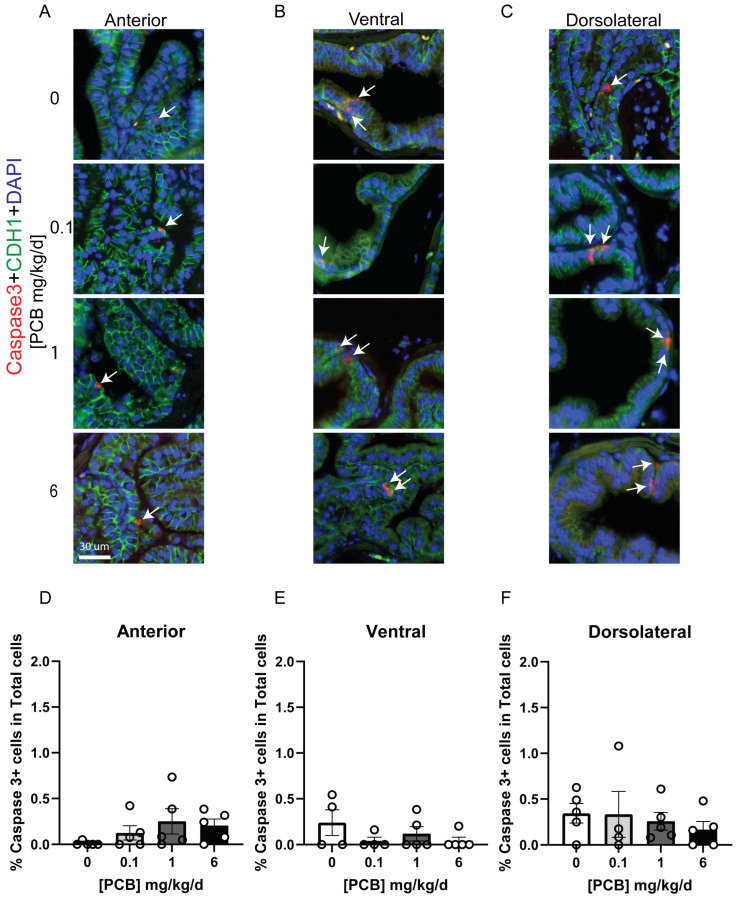
Adult PCB exposure does not alter caspase 3-positive cells in the prostate. Adult male mice were exposed to a daily dosage of the MARBLES PCB mixture for 2 months and tissues were collected for histology. Representative images of (**A**) anterior prostate, (**B**) ventral prostate, and (**C**) dorsolateral prostate stained with antibodies targeting cleaved caspase 3 (red) and E-cadherin (green, all epithelium) and counterstained with DAPI to label all nuclei (blue). Quantification of the percentage of total cells which are caspase3+ in the (**D**) anterior prostate, (**E**) ventral prostate, and (**F**) dorsolateral prostate. Arrows indicate positive cells. Results are mean ± SEM, n = 4–5 per treatment group. No significant differences *p* ≤ 0.05, as determined by a Kruskal–Wallis test.

**Figure 7:**
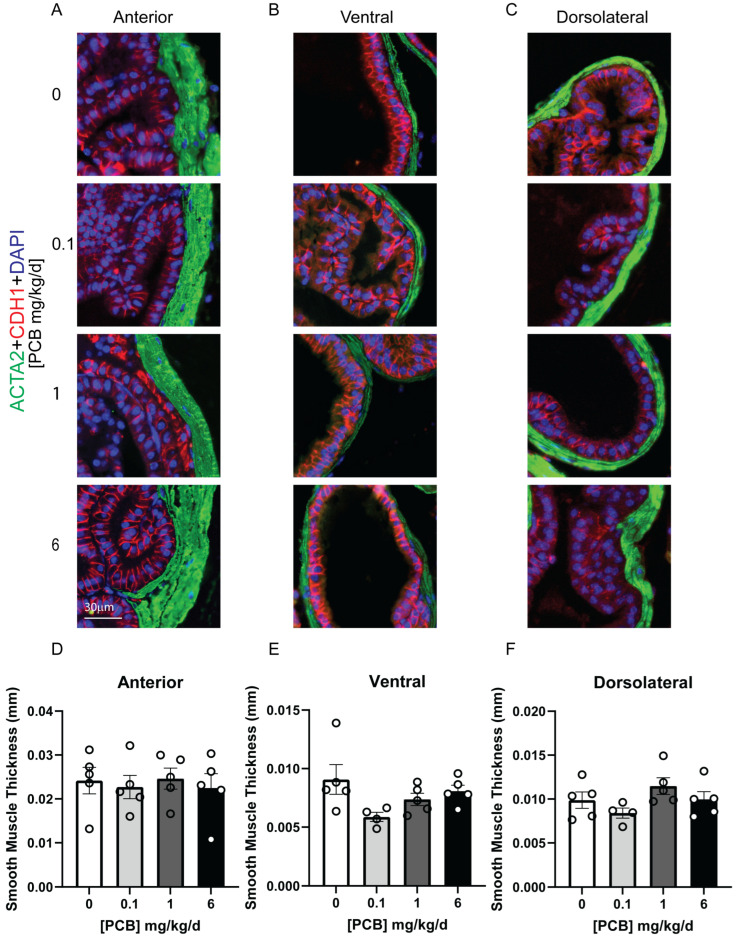
Adult PCB exposure does not alter smooth muscle actin thickness in the prostate. Adult male mice were exposed to a daily dosage of the MARBLES PCB mixture for 2 months and tissues were collected for histology. Representative images of (**A**) anterior prostate, (**B**) ventral prostate, and (**C**) dorsolateral prostate stained with antibodies targeting alpha smooth muscle actin 2 (ACTA2, green) and E-cadherin (red, all epithelium) and counterstained with DAPI to label all nuclei (blue). Quantification of smooth muscle thickness surrounding ducts in the (**D**) anterior prostate, (**E**) ventral prostate, and (**F**) dorsolateral prostate. Results are mean ± SEM, n = 4–5 per treatment group. No significant differences *p* ≤ 0.05, as determined by a one-way ANOVA test.

Aside from assessing gross changes in mass, we sought to determine whether more subtle histological changes were present in the prostate following adult PCB exposure. We first assessed whether PCBs altered the number of cells expressing the cell cycle marker Ki-67. Interestingly, there was a significant dose-specific non-monotonic decrease in the percent of total cells that were Ki-67 positive in the 1 mg/kg/d PCB group versus the vehicle in the anterior and ventral prostate lobes (Figure 5A–E; Anterior F(3,16) = 3.530, *p* = 0.0391, 0 vs. 1 *p* = 0.0424; Ventral F(3,14) = 3.923, *p* = 0.0317, 0 vs. 1 *p* = 0.0151), but not the dorsolateral prostate (Figure 5F). We also assessed whether the number of cells undergoing apoptosis, measured by expression of caspase 3 (Casp3)-positive cells, was altered by PCB exposure. The number of Casp3-positive cells was not significantly altered by PCBs (Figure 6). We also assessed whether the average thickness of the smooth muscle cell layer around individual prostate ducts was altered by PCBs, and there were no significant changes in any prostate lobe (Figure 7). Together, these data indicate that while changes in prostate mass were not seen, subtle histological changes were present with a reduction in proliferating cells only in the 1 mg/kg/d PCB exposure group.

## 4. Discussion

Previous studies have shown that in utero and lactational MARBLES PCB exposure changes voiding physiology and behavior in developing offspring [40]. However, the consequences of PCB exposure in adulthood and the mechanisms underlying these phenotypes are largely unknown. In this study, we demonstrate that a two-month MARBLES PCB exposure in male mice at an adult timepoint minimally changes voiding function but does impact prostate proliferation. The consequences of these changes are unknown but could influence prostate health and voiding function and warrant further study. Additionally, this study helps to advance the field by providing context into the effects of timing and critical windows of PCB exposure and effects on the lower urinary tract.

Results from VSA testing indicated that there were no significant changes in total urine spot number or size between each of the PCB dose groups. About 5% of six-week-old and 10% of nine-week-old male mice void in what is termed a ‘frequent spotter’ phenotype, defined as 50+ and 100+ spots, respectively, during a 4 h VSA [57]. We also found previously that developmental exposure to MARBLES PCB mix at the 0.1 and 6 mg/kg/d dose resulted in young adult offspring being more likely to have a frequent spotter pattern at a younger age [40]. While there were no significant differences in the current study between the percentage of animals with the specified spot numbers during VSA, it is noted that a trend was seen in that the PCB groups all had the same or higher percentages of mice displaying 300 or 400+ urine spots versus the vehicle control. Whether this would become statistically significant over time either as a factor of age and/or continued PCB dosing remains to be determined but raises the hypothesis that adult PCB exposure could influence LUTD.

PCBs often display unique dose effects. This is also observed here, with the results of the uroflowmetry testing showing a decrease in void duration in the 6 versus the 1 mg/kg/d PCB treatment group. While not different from the vehicle control, these results indicate that there are dose-specific effects within PCB exposure groups. Decreased void duration in the context of LUTS could indicate a faster or smaller void; however, void mass and flow rate were not significantly changed at this timepoint. It raises the hypothesis that void duration may be the first parameter altered that could lead to changes in flow rate over time; this is an area of future study. The fact that this endpoint was also specific to the 6 vs. 1 mg/kg/d doses is important to consider, as a non-monotonic dose response is often observed in PCB toxicology studies in that lower doses do not necessarily predict effects of higher or even middle doses. Here, we also observe that the 1 mg/kg/d PCB group has unique effects at this timepoint compared with the other doses assessed. This is not unique; previous studies in developmental exposure models have shown that the 1 mg/kg/d MARBLES PCB mixture leads to adult male offspring having the fewest voiding parameters changed compared with the 0.1 or 6 mg/kg/d treatment groups [40]. This is observed even though PCB levels in the liver, bladder, blood, and urine of these developmentally exposed animals dose-dependently increase in the 0.1, 1, and 6 mg/kg/d PCB dose groups [60]. In previous work relating to the prostate, the 1 mg/kg/d PCB dose was the only treatment group that showed a decrease in ventral prostate mass versus the low or high PCB groups, but not versus the vehicle control, and a decrease in prostate collagen density [41]. Thus, structural prostate effects appear to be present at the 1 mg/kg/d PCB dose, but prostate effects do not necessarily co-occur with voiding effects. This was also true in this study; while prostate mass did not differ, decreases in Ki67-positive cells in the anterior and ventral prostate only occurred in the 1 mg/kg/d PCB group versus vehicle control. This indicates that PCB effects on the prostate were present without overt changes in voiding function. Together, these data help to identify the effects of PCB dose and timing on the function and histological changes of the lower urinary tract. The functional consequence of reduced prostate proliferation is unknown and is an area of future study. While this dosing paradigm was chosen to mimic those of other studies that examined urology endpoints, a limitation of this study is that levels of PCBs were not quantified, and it was not a goal of this work to correlate any effects with exact body burden levels of PCBs. In previous adult studies in non-pregnant female mice dosed for 7 weeks with the same mixture and doses used here, a dose dependent increase in PCBs in the feces, liver, serum, and brain was observed [54,61]. The same was true for post-weaning dams and PCB levels in the intestinal content [62]. The concentration of PCBs in these animals is an area of future study.

This study allows for two critical comparisons with previous studies: 1) the impacts of timing of PCB exposure on voiding and prostate metrics and 2) sex effects following adult PCB exposure. First, in male mice, the timing of PCB exposure influences the physiological outcome. In a previous study, developmental exposure to MARBLES PCB mixture (at the same doses used here) led to male offspring having an increased number of small urine spots and increased void pressures, while prostate mass was reduced only in the 1 mg/kg/d PCB group compared with other PCB groups but not with the vehicle control, with no changes in prostate proliferation observed [40,41]. In contrast, in this study, adult two-month exposure to MARBLES PCB resulted in no changes in voiding, no changes in prostate mass, but a significant decrease in proliferating prostate cells. Taken together, these data illustrate that exposure to MARBLES PCBs has a greater impact on voiding in the developmental stage compared with the adult stage in male mice; however, prostate effects are present under both exposure windows, with mass affected by developmental exposure and proliferation affected by adult exposure. Second, we are able to compare sex effects of adult exposure to MARBLES PCB in relation to voiding function. In a previous study, adult female mice given MARBLES PCB to generate offspring were also tested for voiding function, and they displayed a significant increase in the number of medium-size urine spots in the 1 mg/kg/d PCB group versus the vehicle with no changes seen via uroflowmetry or anesthetized cystometry [45]. Here, the adult males had no changes to voiding assessed via VSA. These data suggest that adult exposure to MARBLES PCB may impact voiding slightly more in female versus male mice. However, the females used in that study were used to generate litters, so they had the added confounding variables of pregnancy and lactation. Whether the adult PCB exposure in a naive non-pregnant female for two months would have similar effects is unknown. While no changes were observed via cystometry, it should be noted that animals are anesthetized for this treatment, which can impact voiding; whether awake cystometry results would differ is unknown, but considering the lack of effects observed in VSA and uroflow (which do not require anesthesia), it is unlikely that dramatic differences would be seen between anesthetized and awake cystometry. Collectively, these comparisons highlight that development is a critical window in establishing adult voiding function, the prostate can be influenced by both developmental and adult exposures, and that among adult exposures, sex effects are present.

The prostate is a hormonally sensitive organ [47] and is sensitive to environmental endocrine-disrupting chemicals such as bisphenyl A, S, and F, which have been shown to act similarly to estrogen and produce a phenotype of increased prostate mass, bladder mass, and an obstruction phenotype of altered voiding function [48,49]. Several PCBs and their metabolites can act as endocrine disruptors [15,21,50,51]. However, here we did not see increases in prostate mass; instead, we observed a decrease in prostate proliferation maker Ki67 in the 1 mg/kg/d PCB group versus the vehicle control. The same pattern is seen in developmental exposure models in which adult offspring show no increase in prostate mass; instead, prostate mass is unchanged relative to the vehicle control or even reduced compared with other PCB doses [41,52]. This suggests that the MARBLES PCB mixture used does not lead to increased prostate mass, as is seen with estrogenic compounds, and likely suggests that this mixture does not promote prostatic growth. This clarifies that prostate enlargement, which is seen in some environmental exposure/hormone models of urinary tract dysfunction and obstruction, is not seen here.

We observed a decrease in Ki67-positive cells in the 1 mg/kg/d PCB group compared with the vehicle control in the anterior and ventral prostate, but not the dorsolateral prostate. These differences may in part reflect known differences in the mouse prostate lobes, which could influence regional responses to endocrine-disrupting chemicals. The ventral prostate is generally considered the most sensitive to androgens, with greater regression following castration compared with the dorsal and lateral lobes [63,64]. Sequencing studies further demonstrate that individual prostate lobes contain distinct cellular signatures and androgen-responsive transcriptional programs with unique luminal epithelial populations identified in the ventral prostate lobe [65]. Furthermore, aging studies in mice report reduced androgen receptor expression in the anterior and ventral but not dorsolateral prostate, and in these same mice, Ki67-positive cells were reduced in all three prostate lobes in aged versus young mice [66]. Together, these studies provide evidence that hormone sensitivity of the prostate lobes could contribute to the unique PCB effects on the ventral and anterior but not dorsolateral prostate lobes. PCBs have been reported to both agonize and antagonize androgen receptor-mediated transcription [67]. Exposure to a different PCB mixture than the one used here was shown to reduce androgen receptor expression in the ventral prostate of adult rats [68]. Because androgen receptor signaling can regulate cell cycle progression [69], it is possible that PCB interference with androgen receptor signaling could alter cell cycle regulators and reduce proliferation. Whether this is occurring here is unknown but an intriguing area of future study. Studies in aging models showing reduced prostate proliferation [66] also raise the possibility that PCB exposure may partially mimic aspects of an aging phenotype, a hypothesis that warrants future investigation.

PCBs are broadly categorized as dioxin-like or non-dioxin-like based on their structure and function [70]. The dioxin-like PCBs are capable of binding the aryl hydrocarbon receptor and act similarly to the environmental chemical dioxin (TCDD). The MARBLES PCB mixture used here only contains one dioxin-like PCB (PCB 118). However, this is of interest since dioxin exposure has also been implicated in altered voiding function and prostate health, with timing of exposure being critical to the observed adverse effects. Developmental exposures to TCDD tend to lead to adult male mice displaying decreased void interval and increased prostate smooth muscle nerve density, which functionally increases prostatic contractile tone [71]. When developmental TCDD exposures are combined with a hormone-induced (T + E2) model of LUTD in mice genetically prone to prostate hyperplasia, TCDD exacerbates increases in prostate mass, proliferation, smooth muscle thickness, and urine retention [72,73]. Conversely in humans, dioxin exposure has been associated with decreased odds of men developing benign growth of the prostate, BPH [74,75,76]. Thus, timing of dioxin exposure likely influences effects on the lower urinary tract. Interestingly, the inverse association of adult dioxin exposure and BPH observed in men is consistent with our findings that adult MARBLES PCB exposure leads to decreased prostate proliferation in mice; additional study will be necessary to test these new hypotheses.

## 5. Conclusions

Together, these studies illustrate that timing is likely critical in assessing the effects of environmental chemicals such as dioxin and PCBs on prostate and LUTS. Furthermore, these phenotypes may be influenced by the genetic substrate or additional aging stressors [73,74,75,76]. Future studies to identify the mechanisms underlying the effects of these chemicals on the lower urinary tract, and the critical windows upon which they act, may help future prevention and therapeutic strategies and help to understand individual risk for LUTS.

## Figures and Tables

**Table 1:** Antibodies used in this study.

Primary Antibodies	Source	Company	Catalog Number	Dilution	
E-Cadherin (CDH1)	Mouse	BD Transduction Labs (via Fisher)	610181 RRID: AB_397581	1:250	
Cleaved Caspase (Casp3)	Rabbit	Cell Signaling	9661 s RRID: AB_2341188	1:200	
Ki67	Rabbit	Abcam	AB15580 RRID: AB_443209	1:200	
Smooth Muscle Actin (Acta2)	Goat	Fisher Scientific	PA518292 RRID: AB_10980764	1:250	
**Secondary Antibodies**					**Pairing**
Anti-Mouse Alexa Fluor 594	Donkey	Jackson ImmunoResearch	715-585-150 RRID: AB_2340854	1:250	CDH1
Anti-Mouse Alex Fluor 488	Donkey	Jackson ImmunoResearch	715-545-150 RRID: AB_2340846	1:250	CDH1
Anti-Rabbit Alex Fluor 488	Donkey	Jackson ImmunoResearch	711-545-152 RRID: AB_2313584	1:250	Ki67
Anti-Rabbit Alexa Fluor 594	Goat	Jackson ImmunoResearch	111-585-144 RRID: AB_2307325	1:250	Casp3
Anti-Goat Alexa Fluor 488	Donkey	Jackson ImmunoResearch	705-545-003 RRID: AB_2340428	1:250	Acta2

**Table 2:** PCB effects on frequent spotting.

Total Urine Spot Number Distribution	mg/kg/d PCB			
	0	0.1	1	6
% mice with over 50 spots	100	75	87.5	87.5
% mice with under 50 spots	0	25	12.5	12.5
% mice with over 100 spots	87.5	62.5	75	75
% mice with under 100 spots	12.5	37.5	25	25
% mice with over 200 spots	50	37.5	25	37.5
% mice with under 200 spots	50	62.5	75	62.5
% mice with over 300 spots	12.5	25	12.5	12.5
% mice with under 300 spots	87.5	75	87.5	87.5
% mice with over 400 spots	0	12.5	0	12.5
% mice with under 400 spots	100	87.5	100	87.5

No significant differences in percent of total mice with indicated spot number between control or any PCB group, as determined by Fisher’s exact test, *p* < 0.05.

## Data Availability

The original data presented in the study are openly available in the Dryad database at DOI: 10.5061/dryad.fttdz096z.

## Abbreviations

The following abbreviations are used in this manuscript:

LUTD	Lower urinary tract dysfunction
PCBs	Polychlorinated biphenyls
LUTS	Lower urinary tract symptoms
BPH	Benign prostatic hyperplasia
